# Microsatellite markers reveal low levels of population sub-structuring of *Plasmodium falciparum* in southwestern Nigeria

**DOI:** 10.1186/1475-2875-13-493

**Published:** 2014-12-13

**Authors:** Muyiwa K Oyebola, Emmanuel T Idowu, Haddy Nyang, Yetunde A Olukosi, Olubunmi A Otubanjo, Davis C Nwakanma, Samson T Awolola, Alfred Amambua-Ngwa

**Affiliations:** Parasitology and Bioinformatics, Faculty of Science, University of Lagos, Lagos, Nigeria; Malaria Research Group, Nigerian Institute of Medical Research, Lagos, Nigeria; Medical Research Council, Gambia Unit, Fajara, The Gambia

**Keywords:** Genetic diversity, *Plasmodium falciparum*, Linkage disequilibrium, Population structure, Genetic differentiation

## Abstract

**Background:**

Genetic diversity studies provide evidence of *Plasmodium falciparum* differentiation that could affect fitness and adaptation to drugs and target antigens for vaccine development. This study describes the genetic structure of *P. falciparum* populations in urban and rural sites from southwestern Nigeria.

**Methodology:**

Ten neutral microsatellite loci were genotyped in 196 *P. falciparum* infections from three localities: Aramoko-Ekiti, a rural community; Lekki, an urban location and Badagry, a peri-urban border settlement. Analysis was performed on the genetic diversity, linkage disequilibrium, population structure and inter-population differentiation.

**Results:**

Allelic diversity values were similar across all populations, with mean expected heterozygosity (H_E_) values between 0.65 and 0.79. No matching multilocus haplotypes were found and analysis of multilocus LD showed no significant index of association. Genetic differentiation between populations was low (ΦPT = 0.017).

**Conclusion:**

The absence of detectable population structure of *P. falciparum* in southwestern Nigeria is evident in the lack of significant differentiation between populations separated by about 200 km. This implies that a fairly uniform malaria control strategy may be effective over a wide geographic range in this highly endemic region. However, more wide-scale survey across the country will be required to inform malaria control in this large and densely populated endemic region.

## Background

The incidence of malaria infections and malaria related mortality has reduced in many countries in Africa [1–3]. However, these successes remain limited in geographical coverage while transmission continues in some endemic regions in sub-Saharan Africa despite concerted efforts to reduce or eliminate the disease [4, 5]. This is partly due to genetic diversity of the main agent *Plasmodium falciparum* which maintains population fitness against targeted interventions such as drugs [6, 7]. Information on genetic diversity and parasite population trends that could help guide control programmes is lacking in regions with large human populations at risk such as Nigeria. The most recent report on patterns of malaria endemicity in Nigeria continues to show high levels of burden across the country with ~170 million people at risk [8]. This is despite more than a decade of vector control with insecticide-treated nets/long-lasting insecticidal nets (ITN/LLINs), indoor residual spraying (IRS), larval control and targeting of parasites with intermittent preventive treatment (IPT) and artemisinin-based combination therapy (ACT). With a proposed agenda for malaria elimination, it is important to determine the extent of genetic diversity, transmission intensity and the ultimate population structure of the parasites to support interventions.

There are various approaches to molecular determination of population structure including typing for polymorphic repeats in merozoite surface proteins (MSP 1 and 2) and glutamate rich proteins (GLURP) [9, 10]. Upon these are microsatellite loci which have proven to be particularly useful due to their abundance, putative neutrality and higher levels of polymorphisms [11–13]. With microsatellite markers, strong linkage disequilibrium (LD), low diversity, and extensive population differentiation have been shown in regions with low levels of transmission [4], in contrast to regions with high levels of transmission [14, 15]. In West Africa, increasing diversity and complexity of infections has been described across a malaria endemicity gradient from Mauritania to The Republic of Guinea [13]. This variance in diversity may be due to variation in vector and human hosts as well as population migration between endemic regions and the transition from seasonal to perennial transmission southward to the Atlantic coast [14, 16]. As with other high transmission regions, molecular markers should show limited differentiation between *P. falciparum* populations in Nigeria.

Earlier studies on Nigerian *P. falciparum* strains showed contrasting dominance between subtypes of GLURP [17], and MSP-1 and MSP-2 [18–20] polymorphic repeats. As these antigenic loci are under strong immune selection [21–23], this may represent variance from immune selection or sampling bias between populations.

To provide further insight into current patterns in parasite population, this study determined the extent of genetic diversity of *P. falciparum* isolates from rural, urban and semi-urban settings in southwestern Nigeria where interventions are being intensified. Neutral microsatellite loci of *P. falciparum* isolates from one inland and two coastal communities in southwestern Nigeria were analysed.

## Methods

### Sample collection and DNA extraction

**Figure 1:**
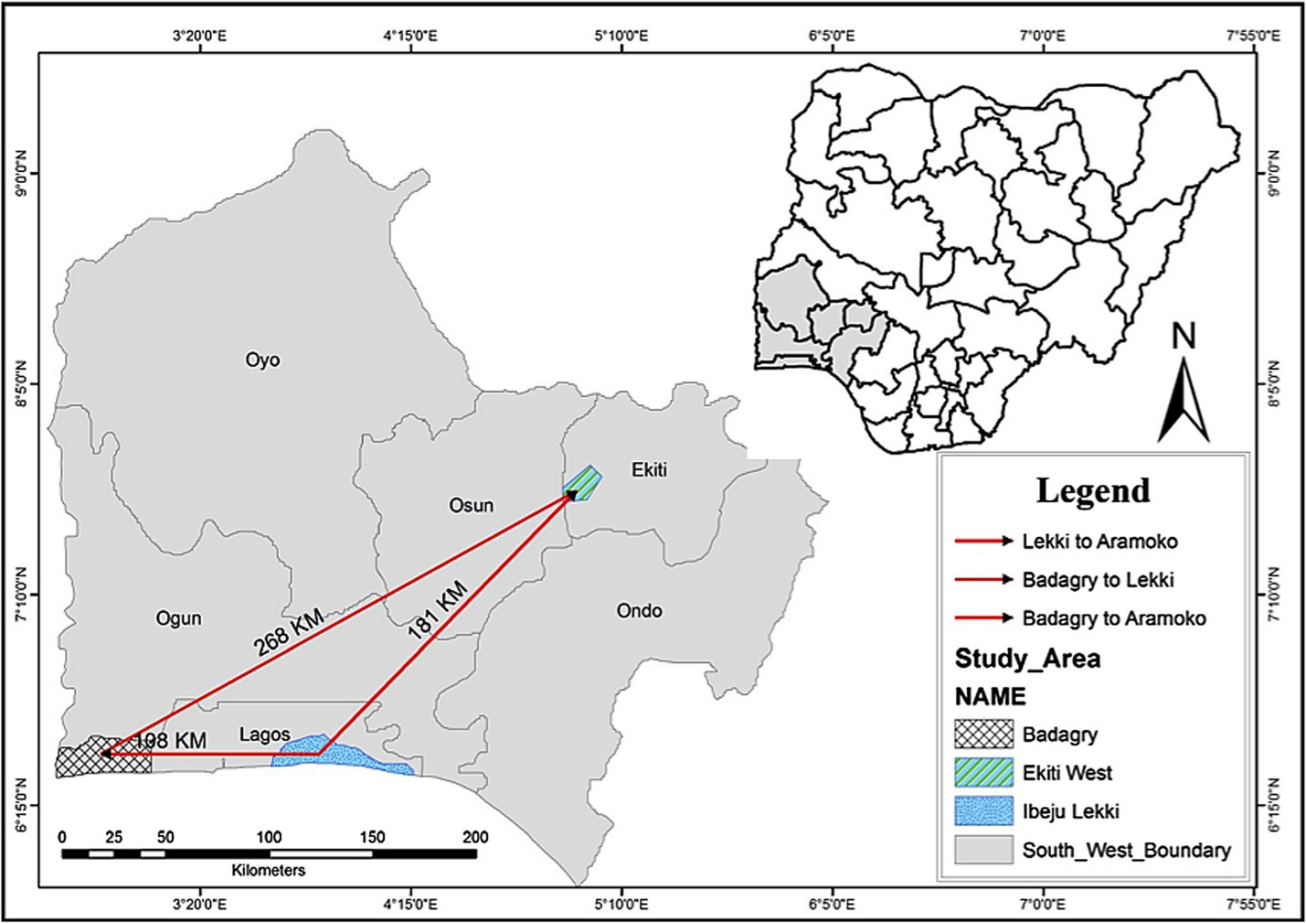
**Map of southwestern Nigeria showing the study areas and the geographic distances between them.** Red lines indicate the distances between sites.

Participants presenting with symptoms of malaria at three health facilities each representing three localities in southwestern Nigeria: Aramoko-Ekiti (AMK), a rural community in Ekiti State; Lekki (LEK), an urban community and Badagry (BDG), a peri-urban border community in Lagos State (Figure 1), were recruited between November, 2012 and December, 2013. All participants or their guardians gave written informed consent to provide blood samples for the study. The study protocols were reviewed by the Institutional Review Board of the Nigerian Institute of Medical Research, Lagos (with reference number IRB/12/209). Thick and thin blood films prepared on microscope slides were stained with 10% Giemsa (v/v) and examined under the microscope (Olympus CX21, UK). *Plasmodium falciparum*-positive samples were spotted on 3 mm Whatmann filter paper (Whatmann International Ltd., Maidstone, UK). Genomic DNA was extracted from punched-out disc from each filter paper dried blood spot using the QIAmp DNA blood midi kit (Qiagen, UK) followed by molecular analyses at The Medical Research Council, Gambia Unit.

### Microsatellite genotyping

A two-round hemi-nested PCR was used to amplify 12 microsatellite loci from parasite DNA following described procedures and primers [24]. The loci included Polyα (Chr4), TA42 (Chr5), TA81 (Chr5), TA87 (Chr6), TA1 (Chr6), TA109 (Chr6), TA40 (Chr10), 2490 (Chr10), ARAII (Chr11), PfG377 (Chr12), PfPk2 (12), and TA60 (Chr13). FAM, HEX and PET-labeled PCR products for different loci amplified from each isolate were pooled together with GeneScan™ 500 LIZ internal size standard (Applied Biosystems, Foster City, CA) for electrophoresis on an ABI 3130XL Genetic Analyzer. Peakscanner (Applied Biosystems) and GeneMarker (Softgenetics) softwares were used for normalization across runs and automatic determination of allele length and peak heights in samples containing multiple alleles per locus, minor alleles were scored when the minor peaks were ≥20% the height of the predominant allele in the isolate and with a relative fluorescent unit of at least 100. Multiple infections were defined when any of the loci contained multiple alleles.

### Population genetic analyses

The allele frequencies, numbers of alleles per locus, allelic diversity within each population, and allele frequencies per locus per population were calculated using GENALEX 6 [24]. Allelic diversity was calculated for each of the microsatellite loci based on the allele frequencies, using the formula for expected heterozygosity, , where n is the number of isolates analyzed and p represents the frequency of each different allele at a locus. H_E_ provides an indication of the probability that two individuals will be different. It has a potential range from 0 (no allele diversity) to 1 (all sampled alleles are different). To understand the potential for multilocus haplotypes to spread through the populations, multilocus linkage disequilibrium (LD) was calculated for the entire population as a whole, and separately for each subpopulation using the standardized index of association, (), using LIAN version 3.5 web interface [25] and the majority allele at each locus in each infection. This index was calculated as () = (1/n – 1 ((V_D_/(V_E_) – 1), where V_E_ is the expected variance of n - the number of loci for which two individuals differ. The observed variance is given by V_D_. To test whether the ratio of V_D_/V_E_ was significantly greater than one, we employed a randomization test as previously described [26, 27].

Between population and within population variance was determined with the analogue of Wright’s Fst, AMOVA (ΦPT), as it is flexible enough to accommodate different types of assumptions about the evolution of microsatellites [28]. ΦPT = 0 was considered indicative of no genetic difference among populations. A distance between isolates from the different populations was estimated in GENALEX 6 which was also employed in implementing a principal coordinate analysis (PCoA) to determine population substructure. Population structure was visualized in an R-dot plot of coordinates 1, 2, and 3.

## Results

**Figure 2:**
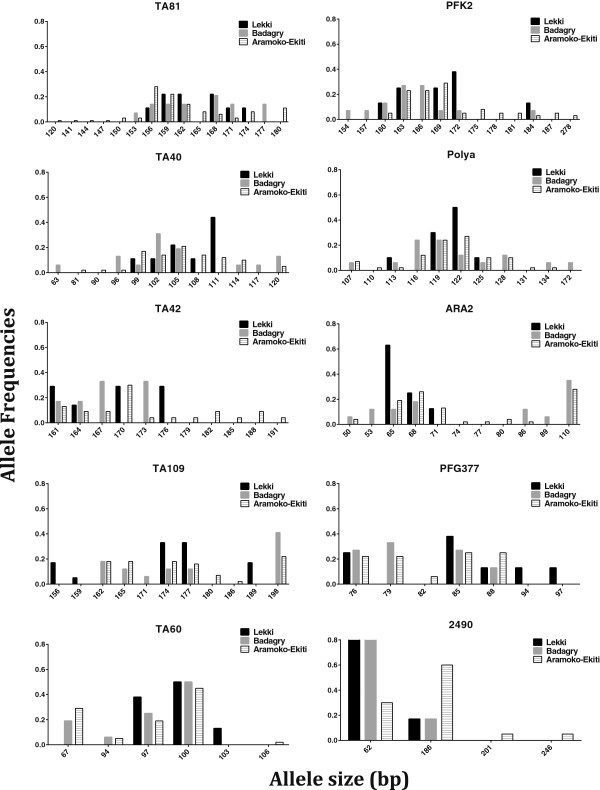
**Frequency distribution of the allele lengths (bp) at 10 microsatellite loci in the three** ***P. falciparum*** **populations.** Allele sizes determined by capillary electrophoresis are shown on the x-axis and their frequencies on the y-axis. Vertical bars for each population represent allele frequencies of major alleles for each microsatellite locus determined using GENALEX 6.0.

A total of 196 isolates of *P. falciparum* infections only were reported. Of the 12 microsatellite loci genotyped, 2 (TA87 and TA1) gave less efficient PCR amplification and were therefore excluded from subsequent analyses. The allelic frequencies at each of the ten loci in each of the three parasite populations are presented in Figure 2. The overall number of alleles per locus observed in the study areas ranged from 8 (for locus 2490) to 27 (for locus TA81). Highest and lowest mean MOIs were recorded in BDG and AMK respectively (Table 1) although the difference across the populations was not significant (P = 0.637). Allelic diversity values were similar across all populations, with mean H_E_ values across all loci between 0.65 (for LEK) and 0.79 (for AMK) (Table 2). Mann–Whitney *U*-test result showed no significant difference in the mean H_E_ values between LEK and BDG as well as BDG and AMK at P < 0.01. However, the difference in the H_E_ values between LEK and AMK was significant (P = 0.01). Although the mean number of genotypes detected per isolate was highest in AMK (Table 3), Kruskal-Wallis test (P = 0.368) showed no substantial difference in the mean number of genotypes in the three parasite populations.

**Table 1:** **Multiplicity of** ***P. falciparum*** **infections in the study areas**

Locus	LEK	BDG	AMK
Poly α	1.60	1.59	1.65
PFPK2	2.00	2.44	2.40
TA81	1.89	2.14	1.96
ARA II	3.13	2.75	2.50
TA40	1.36	1.63	1.43
TA42	1.75	1.50	1.27
2490	1.11	1.33	1.00
TA60	1.18	1.44	1.33
TA109	2.08	1.59	1.47
PFG377	1.46	1.47	1.42
Mean MOI	1.76	1.79	1.64

**Table 2:** **Allelic diversity (H**_**E**_ **) of microsatellite loci from the three parasite populations**

Locus	LEK	BDG	AMK
Poly α	0.66	0.87	0.84
PfPK2	0.73	0.83	0.80
TA81	0.85	0.89	0.83
ARA II	0.55	0.81	0.82
TA40	0.75	0.84	0.91
TA42	0.77	0.77	0.87
2490	0.30	0.30	0.56
TA60	0.62	0.73	0.68
TA109	0.49	0.75	0.83
PfG377	0.78	0.75	0.79
Mean	0.65	0.75	0.79

**Table 3:** **Assessment of the genotypes of each isolate**

Locality	Isolates with given number of genotypes				Mean number of genotypes
	1	2	3	4	
LEK	4	6	0	0	1.60
BDG	9	5	1	1	1.59
AMK	18	18	5	0	1.66

**Figure 3:**
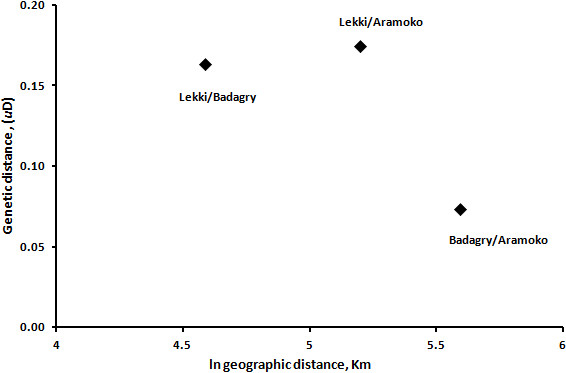
**Relationship between geographic and genetic distances, (** ***u*** **D), for each pair of parasite populations studied.** Genetic distance (y-axis) was determined using GENALEX 6.0 for each pair of populations separated by distance in kilometers (plotted on the x-axis in natural log scale).

**Figure 4:**
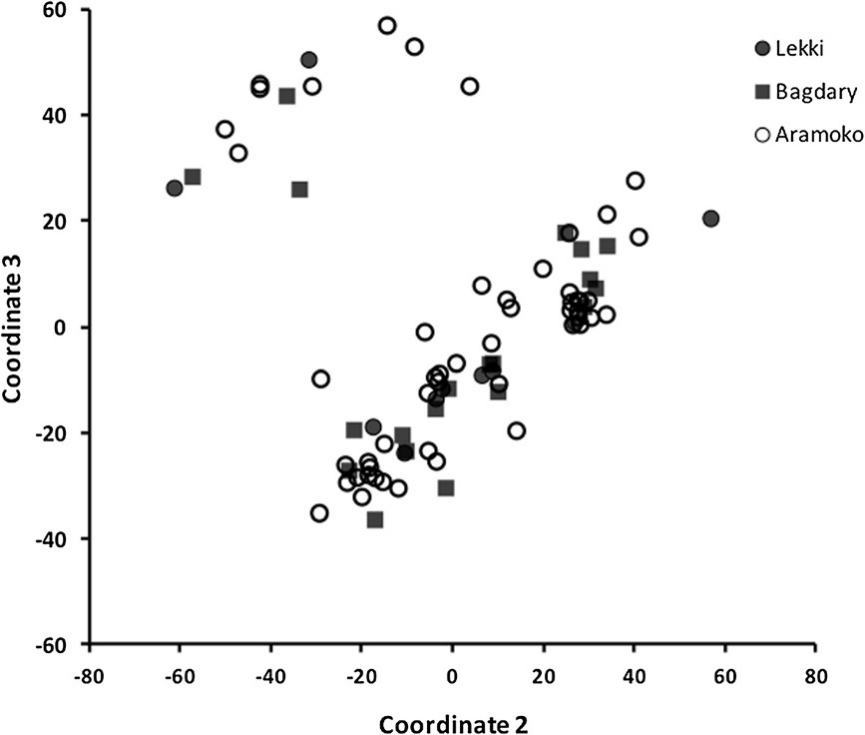
**Population structure of** ***P. falciparum*** **isolates from southwestern Nigeria: Principal coordinate analysis (PCoA) from allelic variance at 10 haploid microsatellite loci.** (Coordinates 2 and 3 in the PCoA show limited sub-structuring of *P. falciparum* isolates into two clusters not determined by site).

Forty-three isolates (~22%) had complete genotype data for all loci from which analysis of multilocus haplotypes was examined. No matching multilocus haplotypes were found. Comparisons of populations using AMOVA showed that genetic differentiation was low with ΦPT = 0.017 (P = 0.772). Pairwise genetic distances between LEK and BDG, LEK and AMK and BDG and AMK parasite populations, calculated as Nei unbiased genetic distance (*u*D), were 0.164, 0.175 and 0.074 respectively. The relationship between genetic distance and the natural log of the geographical distance for each pair of parasite population studied is presented in Figure 3. Principal coordinates analysis (PCoA) showed two distinct clusters of parasites not defined by the origins of individual population (Figure 4). AMOVA also indicated that almost all the genetic variations among parasites (99.98%) were contained within populations. Analysis of multilocus LD showed no significant index of association in all the parasite populations (Table 4).

**Table 4:** **Linkage disequilibrium analysis for each** ***P. falciparum*** **population**

Population	V_D_	V_E_	
LEK	12.174	1.971	0.021
BDG	7.484	1.657	0.032
AMK	4.318	1.544	0.062

## Discussion

Molecular typing of parasite isolates provides vital information about the epidemiological patterns in a population following the implementation of intervention strategies or existence of barriers that could limit gene flow between populations. This study employed microsatellites to determine the structure of *P. falciparum* populations from southwestern Nigeria across an area spanning over 200 kilometres. Samples were recruited from both urban and rural settings to explore parasite population differentiation, given the variation in access to drugs and other interventions [29]. Nigeria is the most populated country in sub-Saharan Africa (sSA) and malaria remains highly prevalent despite varied efforts at interventions. This study is, therefore, timely as the country enters a phase of intervention expansion against malaria.

There was high allelic diversity of 10 microsatellite markers that gave reliable amplification in all the three *P. falciparum* populations analysed. The high expected heterozygosity values were similar to those reported in some other African countries with high levels of malaria transmission [13–15].

Balloux and Lugo-Moulin [30] have put forward that population differentiation values of 0 – 0.05 may suggest low genetic differentiation (GD) among populations; values between 0.05 - 0.15 could indicate moderate differentiation while higher values imply population partitioning into sub-groups. The AMOVA values obtained for the populations sampled were low (0.017) indicating that almost all the genetic variations among parasites (98.3%) were contained within populations. These results were consistent with the low Fst values obtained between the populations. The low levels of genetic differentiation are in agreement with reports from more widely separated but similarly endemic countries in West Africa [13, 14]. Expectedly, they vary from values reported in parasite populations from less endemic Asian [31, 32] and South American [12, 33] countries with similar geographical distances.

The low among population variance and the existence of an inverse relationship in the genetic and geographic distances between BDG and AMK may imply a relatively free gene flow across southwestern Nigeria. Population structure by site of sampling was also not evident by principal coordinate analysis though there was an insignificant sub-grouping distinguishable at the 2^nd^ versus 3^rd^ principal coordinates. Lack of sub-structuring suggests that gene flow precludes local natural selection and genetic drift. This is expected as vector species distribution in southwestern Nigeria is also largely homogenous for *Anopheles gambiae s.s.* negating any possibility of local selection by the vector species [34, 35].

In agreement with previous reports from other high malaria transmission areas [14], there was no significant LD between markers in the three populations owing most likely to the high levels of genetic recombination. Parasites from regions with low prevalence or low levels of multiple infections have been shown to have higher levels of  than those from regions with high prevalence or with high levels of multiple infections [13]. High MOI remains widely reported in Africa particularly among children [36] who constituted the majority of clinical cases in the populations studied. The MOI values obtained varied by marker and site ranging from 1.00 at marker 2490 for AMK to 3.13 for marker ARAII in LEK. As found in other sSA countries, most infections were multi-clonal, with an average MOI of 1.72 across loci and sites in this study. This would favour recombination between genotypes leading to the breakdown of LD, which was low across loci at all sites. High LD could facilitate the spread of drug resistance through transmission of multilocus drug resistance haplotypes [6, 16]. Though linkage was not significant for the entire sampled populations, parasite isolates from AMK with lower mean MOI had higher  value. The AMK site is rural and at a geographic distance of about 200 km from Lagos metropolis. Though the LD seen in AMK remains low compared to South-East Asia [31, 32], it will be important to continue studies in this population and other isolated populations in Nigeria to detect any new patterns that may favour adaptation against interventions.

## Conclusion

This report suggests the population of *P. falciparum* in this region is diverse with an absence of detectable population structure indicating panmixia. The limited differentiation in parasite populations is likely to be a consequence of high and continuous transmission of randomly mating *P. falciparum* isolates facilitated by indiscriminate vector and human migration. A country-wide study of diversity will be needed to support these findings and inform the current drive to deploy interventions towards the elimination of malaria.
